# The omicron variant of SARS-CoV-2 drove broadly increased seroprevalence in a public university setting

**DOI:** 10.1371/journal.pgph.0003893

**Published:** 2025-01-03

**Authors:** Ching-Wen Hou, Stacy Williams, Guillermo Trivino-Soto, Veronica Boyle, David Rainford, Selina Vicino, Mitch Magee, Yunro Chung, Joshua LaBaer, Vel Murugan

**Affiliations:** 1 Virginia G. Piper Center for Personalized Diagnostics, Biodesign Institute, Arizona State University, Tempe, Arizona, United States of America; 2 College of Health Solutions, Arizona State University, Phoenix, Arizona, United States of America; PLOS: Public Library of Science, UNITED STATES OF AMERICA

## Abstract

Omicron is the comparatively most transmissible and contagious variant of severe acute respiratory syndrome-coronavirus-2 (SARS-CoV-2). We conducted a seroprevalence study from March 1–3, 2022, to investigate the seroprevalence of SARS-CoV-2 antibodies among individuals aged 18 years and older after the Omicron outbreak. The seroprevalence of anti-receptor binding domain (RBD) antibodies was found to be 96.3% (95% CI 95.2–97.2%) compared to 88.2% (95% CI 86.1–90%) in our previous serosurvey. For anti-nucleocapsid (NC) antibodies, the seroprevalence was 39.1% (95% CI 36.6–41.7%) compared to 19.7% (95% CI 17.5–22.2%) earlier. Individuals that experienced breakthrough infections exhibited the highest levels of anti-RBD antibodies. Additionally, saliva samples showed promise as a potential diagnostic biofluid for measuring antibody levels, as they exhibited a strong agreement with the data obtained from serum samples. The near doubling of anti-NC reactivity, a proxy for history of infection, reflects the contagiousness of the omicron variant, but may also have been influenced by a more relaxed approach to precautions in the spring of 2022. Serosurveys repeated at regular intervals monitor the trend of infections in the community, delineate the geographical spread of the infection, and may guide containment measures in communities, and prompt response to future outbreaks.

## Introduction

Arizona State University (ASU) was the 6th largest public university in the USA by enrollment, with 79,232 students enrolled during the 2022–23 academic year [1, 2]. It is situated in the southwest region of the USA, with a warm and arid climate, where outdoor activities are counterintuitively more common in the winter than in the summer. In the summer of 2020, Arizona had the highest COVID-19 infection rate worldwide [3]. In response to the COVID-19 pandemic, ASU switched to remote learning to prevent community-based transmission of SARS-CoV-2 during the first year of the pandemic. However, since the development and distribution of COVID-19 vaccines, the university returned to full in-person learning in Fall 2021 [4]. Understanding whether face-to-face learning has contributed to pathogen spread, especially during the Omicron outbreaks, which is the most contagious variant, is crucial for informing future pandemic responses.

ASU has been managing COVID-19 cases since January 2020. It provided a COVID-19 update of known cases in the university community each week. However, most SARS-CoV-2 infections are mild or asymptomatic and may not be detected by surveillance [5, 6], making the infection-to-case ratio a helpful tool for identifying regions with insufficient testing. Experts estimated that for every virologically confirmed case, about ten infections may have been missed by surveillance systems. Population-based serosurveys are valuable for estimating the proportion of the population previously infected with SARS-CoV-2, providing information about the extent of transmission in the past, and helping to understand the future course of the pandemic [7–10].

In 2021, two more transmissible variants of the COVID-19 virus, Delta and Omicron, emerged sequentially. By June 2021, Delta had become the dominant strain worldwide, and it was linked to an uptick in reported school outbreaks. However, in November 2021, Omicron emerged and quickly replaced Delta as the prevailing strain globally by January 2022. The symptoms associated with the Omicron infection are generally less severe compared to the Delta, but Omicron exhibits higher transmissibility and shows reduced susceptibility to vaccines. It is important to note that the mortality rate associated with Omicron was lower than that of other strains [11–13].

The main objective of this study was to measure antibodies against both the RBD and NC proteins using FDA EUA authorized serological assays to estimate the seroprevalence in the university population after the Omicron outbreak. Our comprehensive analysis of this serosurvey provided critical information: a) The extent of COVID-19 exposure among the university population during the Omicron outbreak; b) the proportion of individuals who have received COVID-19 vaccination and booster doses; and c) the duration of detectable antibodies following vaccination or infection. Furthermore, we compared the findings from this serosurvey with the data obtained during the Delta outbreak serosurvey [14]. This comparison allowed us to gain valuable information regarding the infection rate, vaccination rate, and seroconversion rate of these two strains. Additionally, we compared the performance of assays using two different sample types, saliva, and serum, obtained from the same participants, within 30 minutes of each other. Saliva collection is a non-invasive method that allows individuals to self-collect samples at home. However, it has received relatively little attention for detecting antibodies to SARS-CoV-2 antigens due to its lower concentration of antibodies compared to serum samples [15]. The data collected from saliva samples may have implications for potential use in future serosurveys. Overall, these surveys help elucidate the trends in antibody response against SARS-CoV-2 proteins during the ongoing pandemic, thereby contributing to our understanding of both natural and vaccine-induced immunity.

## Materials and methods

### Ethical approval

The study was approved by ASU’s institutional Review Board (IRB)(STUDY00015522).

### Study design and participants

**Fig 1:**
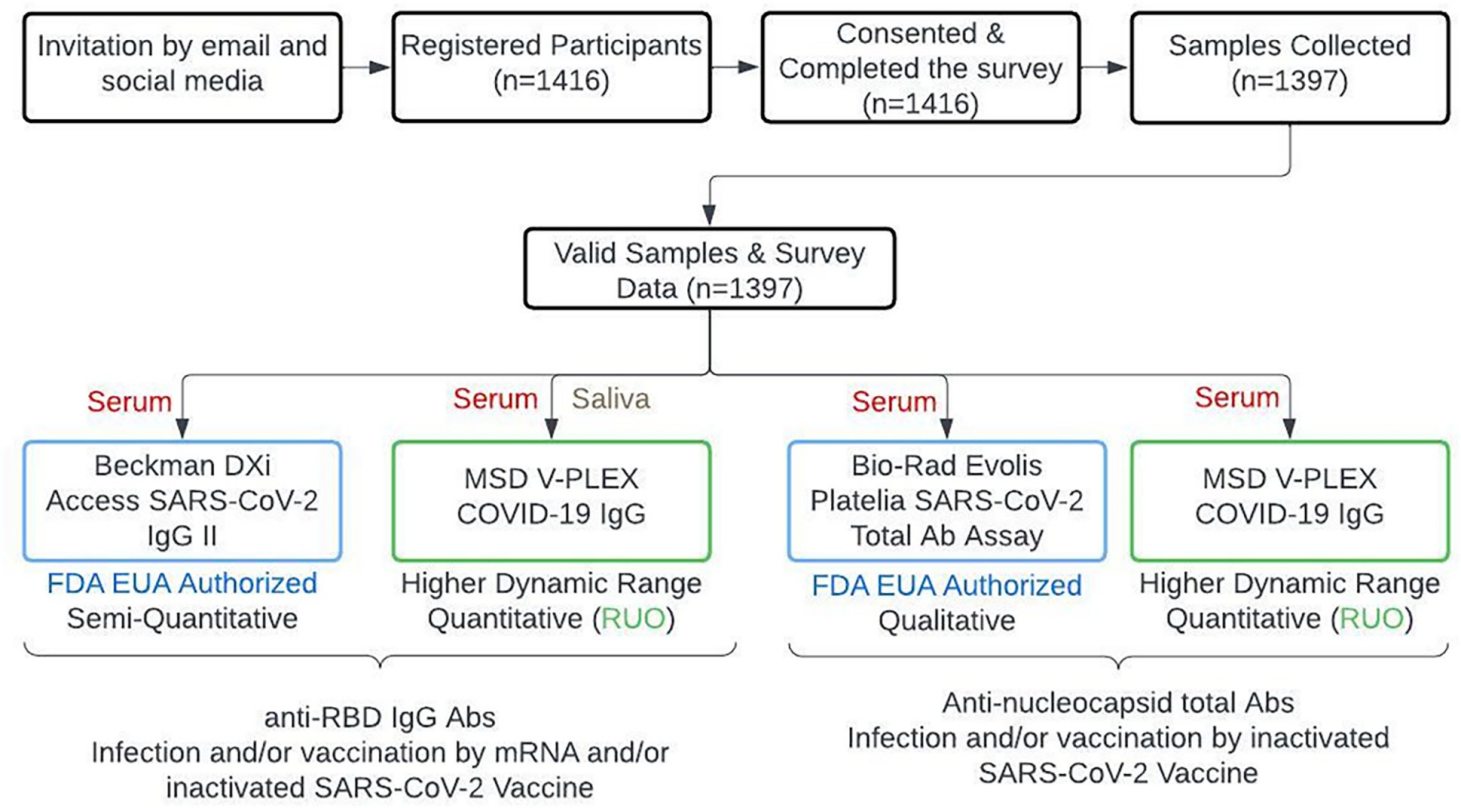
Flowchart of the study design. Recruitment, sample selection, and the assays/specimens used to detect antibody levels.

Recruitment for this study was conducted through invitations, email announcements to the ASU community, and social media advertising, and potential participants were required to complete an electronic consent and a survey before giving biological specimens. The sample collection was extended for three days, from March 1 to March 3, 2022. The study design and sample selection process are shown in Fig 1. Participants were initially invited via emails and social media channels. Individuals were eligible for inclusion if they were 18 years of age or older and were able to provide informed consent. Of the 1416 individuals who completed the screening, provided informed consent, and filled out the initial survey forms, a total of 1397 participants, including ASU students and employees, were successfully recruited for the study. Participants were compensated up to $50 for completing the survey and submitting samples. Individuals under the age of 18, those unable to provide consent, pregnant women, or those weighing less than 110 lbs. at the time of the survey were excluded.

### Participant consent

All participants are 18+ years old and consented to participating in the study and were willing to provide their samples for the research.

### Survey instruments

Demographic information, COVID-19 vaccination status, testing history, and COVID-19 symptoms were collected through a self-reported questionnaire. Participants provided this information voluntarily and compensated after giving the blood and saliva samples.

### Blood sample collection

The blood samples were collected by trained phlebotomists at ASU using serum tubes (Cat # 37988 from BD). Within 4 hours of collection, the samples were placed in a cooler for transportation to the clinical testing laboratory at ASU. Upon arrival, the samples were centrifuged at 1300 g for 20 minutes to separate the serum. A total of 1397 serum samples, along with their corresponding survey results, were included in the analysis.

### Saliva sample kit collection

The saliva samples were self-collected by participants using saliva collection kits. The collection kit was developed by ASU and included a straw, a sterile sample collection tube, a disinfectant wipe, an absorbent pad, and a biohazard bag. Prior to collection, participants were instructed to refrain from eating, drinking, smoking, vaping, chewing gum, brushing their teeth, or using oral hygiene products for at least 30 minutes. Participants were advised to wash and dry their hands before the collection process. To collect the saliva sample, participants were provided with a straw and instructed to use it to fill the tube with saliva until it reached the fill lines indicated (excluding bubbles). Once the desired amount of saliva was collected, participants removed the straw and sealed the tube with the provided cap. After sealing the tube, participants were instructed to wipe it with the provided disinfectant wipe and place it in the biohazard bag, along with the absorbent pad.

### qPCR testing

qPCR testing was conducted at the Clinical Laboratory Improvement Amendments (CLIA) certified, College of American Pathologists (CAP) accredited ASU Biodesign Clinical Testing Laboratory (ABCTL), using EUA authorized, TaqPath COVID-19 Fast PCR Combo Kit (Applied Biosystems, CA, USA; Cat#A51606) as per manufacturer’s instructions. Briefly, 20 μL of salivaReady^TM^ solution was mixed with 20 μL of saliva and incubated for 5 min each at 62°C and 92°C. 14 μL of saliva plus SalivaReady^TM^ solution was mixed with 6 μL of TaqPath 1-Step Master Mix (No ROX) plus TaqPath COVID-19 Fast PCR assay (ORF1a+ORF1b+N gene+RNase P) before subjected to qPCR amplification in QuantStudio 7 Flex instrument.

### Serology testing

In this survey, we used serological assays from three different platforms; 1) an EUA authorized, semi-quantitative Beckman Access SARS-CoV-2 IgG II assay to measure anti-RBD IgG antibodies; 2) an EUA authorized, qualitative Bio-Rad Platelia SARS-CoV-2 Total Ab ELISA assay to measure anti-NC antibodies; 3) quantitative research assay with large dynamic range (3–4+ logs) by Meso Scale Diagnostics to measure anti-RBD and NC IgG antibodies. Serological tests were conducted either at ABCTL (for EUA authorized clinical assays) or at the Center for Personalized Diagnostics (CPD) (for research use only MSD assays). The two EUA authorized assays were used to detect antibodies in serum samples, whereas the MSD platform was added because of its ability to measure antibodies in saliva.

The Access SARS-CoV-2 chemiluminescent IgG II, a semi-quantitative assay employed five different concentrations of calibrators and two different concentrations of controls, supplied by the manufacturer, to ensure the integrity of the reagents and proper performance of the assay prior to analyzing the samples. The results obtained were compared to a cutoff value expressed in arbitrary units (AU/mL), which was established during the instrument calibration process.

The Interpretation of Platelia SARS-CoV-2 Total Ab ELISA assay was based on the guidelines provided by the manufacturer, as part of the instructions for use (IFU), and was authorized by the FDA: values < 0.8 were considered negative, values between > 0.8 and < 1.0 were categorized as equivocal, and values≥1.0 were considered positive.

The Meso Scale Discovery (MSD) coronavirus panel from Meso Scale Diagnostics was a multiplexed immunoassay designed to measure the IgG antibody response to SARS-CoV-2. Each well of the 96-well MSD plate contains different antigens. A calibration curve was established using a reference standard with 4-fold serial dilutions and a zero-calibrator blank for quantitation. The assay also included three levels of controls to ensure the accuracy of the performance. To perform the assay, the plate was first blocked with Blocker A solution for 30 minutes at room temperature (RT). Following three washes with 150 μL/well of MSD wash buffer, 50 μL of calibrator, controls, and diluted samples were dispensed into the plate and incubated with shaking for 2 hours at RT. After incubation and another round of washes, the detection antibody was added and incubated with shaking for 1 hour. Subsequently, the plate was washed with the wash buffer, and reader buffer B was added for reading the plate using the MESO QuickPlex SQ 120 instrument. The multiplexed immunoassay provided quantitative antibody responses to the antigens of interest. The results were reported in AU/mL, as defined during the calibration of the instrument.

### Statistical analysis

We performed descriptive statistics for demographic, vaccination-related, and self-reported previous infection variables (n = 1,397). We implemented a three-part statistical analysis plan. First, we estimated the seroprevalences of anti-RBD IgG and anti-NC total antibodies, the primary outcomes of interest, and compared these by self-reported infection and vaccination statuses using two-sample proportional tests. Only participants with previous infection exhibit antibodies against NC, we further subset the data by those who self-reported infection (n = 529), and their seroconversion outcome variable whether they were NC positive or negative. In addition, we subset the data by those who were fully vaccinated (n = 1242) with the seroconversion outcome variable being whether they were positive or negative from the RBD assay. We used relative risk models to model the ratio of seroconversion probability as a linear function of the demographic variables. We combined the older age groups (41 years and older) in our models due to the small sample size of those above 65 years of age. Second, the secondary outcome of interest was anti-RBD IgG levels. The study initially divided participants into various categories based on their vaccination and infection status, including those that had received only the vaccine, those that had been infected with COVID-19 only, and those that had experienced both. The participants were then further grouped based on the duration between their most recent vaccination or infection date and the collection date, dividing them into four categories: 0–3 months, 4–6 months, 7–9 months, and≥ 10 months. Since the antibody levels were expected to decay after vaccinations or infections, we stratified the samples based on these four categories and compared the antibody levels between vaccinated, boosted, and infected groups using Mann-Whitney tests. We performed a similar serosurvey before the Omicron outbreak and here we compared anti-RBD IgG levels, vaccination, and infection statuses between serosurvey I (After the Delta and before the Omicron outbreak) and II (after the Omicron Outbreak) data to assess if the Omicron impacted the antibody levels, using Mann-Whiteny tests and two-sample proportional test. Third, as the MSD assay would be useful in detecting anti-RBD antibody levels in saliva samples, its performance was compared to the Beckman and Bio-Rad assays. Specifically, anti-RBD antibody levels quantified by the MSD and Beckman assays were evaluated using Spearman’s coefficient correlation and given thresholds explained in the serological testing section above. Its agreement with the Beckman and Bio-Red assays was evaluated using positive and negative percentage agreements (PPA and NPA). P-values less than 0.05 were considered statistically significant. R version 4.2.1 and GraphPad Prism 9.5.1 were used for statistical analysis.

## Results

### Demographics

The survey included 1397 participants from ASU that provided both saliva samples for qPCR diagnostic testing and blood donations. Of these participants, 820 (58.7%) were students, 562 (40.2%) were employees, and 15 (1.1%) did not provide information about their occupational status (Table 1). Among the 1397 participants for whom occupational data were available, 794 (56.8%) were female and 570 (40.8%) were male. Regarding age, 682 participants (48.8%) were 18–25 years of age, 386 (27.6%) were 26–40, 298 (21.3%) were 41–65, 18 (1.3%) were older than 65, and 13 (0.9%) did not report their age (Table 1). Out of the 1397 participants, 37.9% (n = 529) previously reported testing positive for COVID-19, while 61.1% (n = 853) reported no history of a positive test.

**Table 1:** Demographics of study participants.

		Students (n = 820)	Employees (n = 562)	Total (n = 1397)
Gender	Female	409 (49.9%)	381 (67.8%)	794 (56.8%)
	Male	395 (48.2%)	174 (31%)	570 (40.8%)
	Other	16 (2%)	6 (1.1%)	22 (1.6%)
	Not Reported	NA	1 (0.2%)	11 (0.8%)
Age	18–25	625 (76.2%)	57 (10.1%)	682 (48.8%)
	26–40	186 (22.7%)	199 (35.4%)	386 (27.6%)
	41–65	7 (0.9%)	289 (51.4%)	298 (21.3%)
	>65	NA	16 (2.8%)	18 (1.3%)
	Not Reported	2 (0.2%)	1 (0.2%)	13 (0.9%)
Race	White	272 (33.2%)	380 (67.6%)	656 (47%)
	Asian	331 (40.4%)	62 (11%)	393 (28.1%)
	Mixed	74 (9%)	26 (4.6%)	100 (7.2%)
	Black	22 (2.7%)	15 (2.7%)	37 (2.6%)
	Native	3 (0.4%)	6 (1.1%)	9 (0.6%)
	Hispanic or Latino	93 (11.3%)	61 (10.9%)	154 (11%)
	Other	25 (3%)	11 (2%)	37 (2.6%)
	Prefer not to say	NA	NA	NA
	Not Reported	NA	1 (0.2%)	11 (0.8%)
Vaccination Status	Yes	307 (37.4%)	80 (14.2%)	389 (27.9%)
	Yes + booster	483 (58.9%)	448 (79.7%)	934 (66.9%)
	No	29 (3.5%)	32 (5.7%)	61 (4.4%)
	Not Reported	1 (0.1%)	2 (0.4%)	13 (0.9%)
Vaccine Source	Pfizer	361 (44%)	303 (53.9%)	668 (47.8%)
	Moderna	242 (29.5%)	201 (35.8%)	443 (31.7%)
	Janssen	74 (9%)	19 (3.4%)	93 (6.7%)
	AstraZeneca	94 (11.5%)	2 (0.4%)	97 (6.9%)
	Covaxin	12 (1.5%)	NA	12 (0.9%)
	Sinopharm	3 (0.4%)	NA	3 (0.2%)
	Sinovac	1 (0.1%)	NA	1 (0.1%)
	Not Reported	4 (0.5%)	5 (0.9%)	19 (1.4%)
	unvaccinated	29 (3.5%)	32 (5.7%)	61 (4.4%)
previous self-reported COVID-19 infection	Yes	339 (41.3%)	189 (33.6%)	529 (37.9%)
	No	480 (58.5%)	369 (65.7%)	853 (61.1%)
	Not Reported	1 (0.1%)	4 (0.7%)	15 (1.1%)

In the study, 1397 participants were surveyed about their vaccination status. Of these, 879 participants (62.9%) reported being fully vaccinated with a booster, while 1323 participants (94.7%) reported having received at least one dose of the vaccine. Only 61 participants (4.4%) reported never having received a vaccine. Among the vaccinated participants, the majority (47.8%, n = 668/1397) received the Pfizer vaccine, followed by Moderna (31.7%, n = 443/1397) (Table 1).

### Seroprevalence

#### SARS-CoV-2 RBD of spike IgG

All serological assays were evaluated for detecting anti-RBD IgG antibodies using a set of 1397 serum samples. The seroprevalence of anti-RBD IgG antibodies was found to be 96.3% (95% CI 95.2–97.2%), which did not differ significantly across the groups, including all participants, students only, and employees only (Table 2).

**Table 2:** Anti-RBD and Anti-NC antibody seroprevalence status of the population from Serosurvey II.

Antigen Detected	Name of the test	Positives	Negative	Inconclusive
RBD (IgG)	Access SARS-CoV-2 IgG II	1346 (96.3%) (95% CI 95.2–97.2%)	51 (3.7%) (95% CI 2.8–4.8%)	0 (0%) (95% CI 0–0.3%)
NC (Total Ab)	Platelia SARS-CoV-2 Total Ab Assay	546 (39.1%) (95% CI 36.6–41.7%)	823 (58.9%) (95% CI 56.3–61.5%)	28 (2%) (95% CI 1.4–2.9%)

Out of the 491 participants that self-reported a history of COVID-19 infection and vaccination (excluding four participants that received attenuated parasite vaccines and three participants that didn’t provide the source of their vaccine), 488 (99.4%) tested positive for anti-RBD antibodies. Among the 30 participants that self-reported a history of COVID-19 infection and were not vaccinated, 21 (70%) tested positive for anti-RBD antibodies (*p<0*.*001*, two-sample proportion test). Of the 820 participants that self-reported no history of COVID-19 infection and were vaccinated, 795 (97%) tested positive for anti-RBD antibodies. Among the 31 participants that self-reported no history of COVID-19 infection and were not vaccinated, 18 (58.1%) tested positive for anti-RBD antibodies (*p<0*.*001*, two-sample proportion test) (Table 3).

**Table 3:** Cohort characteristics and serological positive results by different assays from Serosurvey II.

Cohort			RBD Protein	NC Protein
			IgG	Total ab
Infection	Vaccine	n	Beckman	ELISA
Yes (n = 529)	YES^A^	491	488 (99.4%) (95% CI 98.2–99.8%)	348 (70.9%) (95% CI 66.7–74.7%)
	YES^B^	4	4 (100%) (95% CI 51–100%)	4 (100%) (95% CI 51–100%)
	YES*	3	3 (100%) (95% CI 43.9–100%)	3 (100%) (95% CI 43.9–100%)
	NO	30	21 (70%) (95% CI 52.1–83.3%)	25 (83.3%) (95% CI 66.4–92.7%)
	NA	1	1 (100%) (95% CI 20.7–100%)	1 (100%) (95% CI 20.7–100%)
No (n = 853)	YES^A^	805	781 (97%) (95% CI 95.6–98%)	133 (16.5%) (95% CI 14.1–19.2%)
	YES^B^	12	11 (91.7%) (95% CI 64.6–98.5%)	10 (83.3%) (95% CI 55.2–95.3%)
	YES*	3	3 (100%) (95% CI 43.9–100%)	2 (66.7%) (95% CI 20.8–93.9%)
	NO	31	18 (58.1%) (95% CI 40.8–73.6%)	12 (38.7%) (95% CI 23.7–56.2%)
	NA	2	1 (50%) (95% CI 9.5–90.5%)	0 (95% CI 0–0%)
NA (n = 15)	YES	5	5 (100%) (95% CI 56.6–100%)	2 (40%) (95% CI 11.8–76.9%)
	NA	10	10 (100%) (95% CI 72.2–100%)	6 (60%) (95% CI 31.3–83.2%)
Total		1397	1346 (96.3%)	546 (39.1%)

A: Pfizer, Moderna, AstraZeneca, and Janssen; B: Covaxin, Sinopharm

*: Participants did not provide vaccine source

#### SARS-CoV-2 NC antibodies and acute infection status

Overall, the seroprevalence for total anti-NC was 39.1% (95% CI 36.6–41.7%) (Table 2). Out of the 529 participants that reported having had COVID-19, regardless of their vaccination status, 381 (72%) tested positive for anti-NC antibodies (Table 3). Among the 836 participants that reported no previous history of COVID-19 (excluding 12 participants who received attenuated parasite vaccines, three that did not provide vaccine information, and two that did not report their vaccination status), 17.3% (n = 145) tested positive for anti-NC antibodies, suggesting they had had a previous, undetected COVID-19 infection (Table 3).

We also investigated the prevalence of PCR positivity for active viral infection in 1397 asymptomatic students and employees from a university community on the days of sample collection to understand the potential role of asymptomatic carriers in COVID-19 outbreaks in the community, particularly during the Omicron outbreak and found it to be 0.4% (n = 6).

### Seroconversion analysis

Seroconversion refers to the timepoint when antibodies against a virus are detected in the blood following viral infection or vaccination. Our findings indicated that there were no significant differences observed among different races, age groups, gender, and employment status in terms of their ability to generate anti-RBD antibodies following self-reported vaccination. Interestingly, our analyses also indicated that White participants were less likely to seroconvert anti-NC antibodies following self-reported exposure to the SARS-CoV-2 virus compared to Others (prevalence ratio (PR) = 0.87 (95% CI 0.77–0.99), p = 0.03) and Asian individuals (PR = 0.83 (95% CI 0.73–0.94), p<0.01). In addition, individuals 26–40 years of age were less likely to seroconvert anti-NC antibodies than those 18–25 years of age (PR = 0.82 (95% CI 0.71–0.95), p = 0.01). Interestingly, those 41 years and older were more likely to seroconvert anti-NC antibodies than those 26–40 years old (PR = 1.23 (95% CI 1.01–1.49), p = 0.04) (S1 Table).

### Anti-RBD IgG antibody levels after vaccination

**Fig 2:**
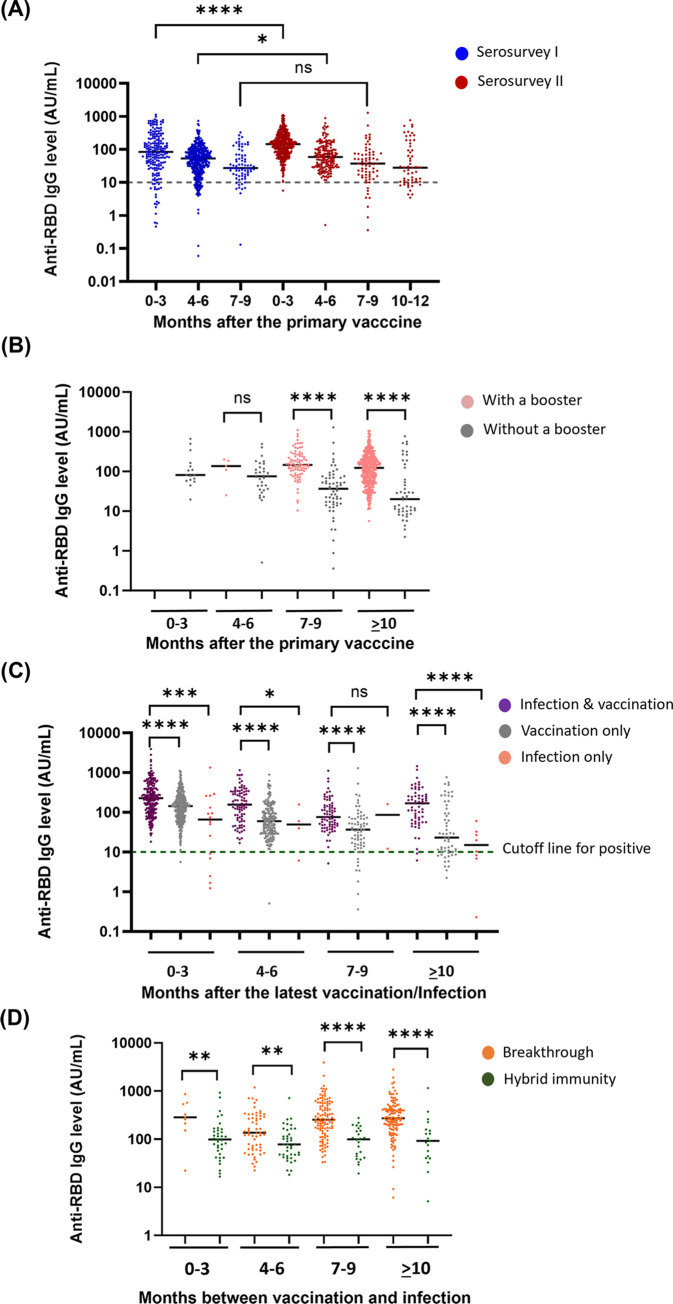
Antibody response in participants with previous COVID-19 infection, vaccination, or both. Anti-RBD antibodies were measured in **(A)** participants that had previous COVID-19 vaccines without self-reported prior infection. **(B)** Participants that did not have a prior infection and were divided into subgroups based on whether they had received a booster or not. **(C)** Participants that had a previous COVID-19 infection or COVID-19 vaccines or both. Participants were categorized by the vaccine or COVID-19 infection and the time interval from vaccination/infection to blood sample collection. **(D)** Participants were categorized based on the order and approximate time scale of COVID-19 infection and vaccination for each group. Cutoff defined per manufacturer. *P values are calculated by the Mann-Whitney test. *p-value <0.05, **p-value <0.01, ***p-value <0.001 and ****p-value <0.0001.

In our study, we investigated the persistence of anti-RBD antibody titers among participants that received a COVID-19 vaccination without self-reported prior infection. The X-axis represents the number of months after the primary vaccination (second dose of vaccine or one dose for Janssen vaccine), while the Y-axis indicates the anti-RBD IgG level in Fig 2A. Our findings revealed that antibodies could still be detected 12 months after vaccination (shown in red in Fig 2A). Additionally, we also compared these findings with the data obtained from Serosurvey I conducted in September 2021 (shown in blue in Fig 2A). Both serosurveys demonstrated a similar trend of declining anti-RBD antibody levels over time. For Serosurvey I, the median anti-RBD antibody level among participants three months after vaccination was 84.42 AU/mL (95% CI 63.88–102.12). In Serosurvey II, the corresponding value was 143.96 AU/mL (95% CI 134.26–156.42). Notably, the most substantial decline occurred between three months and the 4–6-month post-vaccination period in both Serosurveys (*p*<0.0001 for both). Subsequently, antibody levels continued to decrease, reaching 27.2 AU/mL (95% CI 19.23–29.94) and 37.09 AU/mL (95% CI 23.23–49.33) for participants 7–9 months after vaccination in Serosurvey I and Serosurvey II, respectively. The median level of anti-RBD antibodies among vaccinated participants in Serosurvey II was significantly higher compared to Serosurvey I, particularly within the 0–3 months post-primary vaccination. Next, some participants had received a booster previously, while others did not. Therefore, we divided the vaccine group into subgroups: with or without a booster (Fig 2B). After the primary vaccine, in the group without a booster, the median level of anti-RBD IgG antibodies decreased over time (shown in grey dots), while the median level remained constant in the group with a booster (shown in pink dots). The median level of anti-RBD antibodies among booster-vaccinated participants in Serosurvey II was significantly higher compared to those without a booster at 7–9 months (145.55 AU/mL (95% CI 111.84–164.48) vs 36.73 AU/mL (95% CI 22.50–51.77), p<0.0001, and≥10 months (122.49 AU/mL (95% CI 107.63–136.63) vs 20.12 AU/mL (95% CI 10.68–28.63), p<0.0001.

### RBD antibody responses following vaccination and infection

Among all groups, the median level of anti-RBD antibody levels was found to be higher in the subgroups of vaccinated participants that had also been infected with COVID-19 compared to those that had received only the vaccine or had only been infected. Notably, the participants that had never been vaccinated (infection only) trended for lower median anti-RBD antibody levels after infection, contrasting with the hybrid group (vaccination and infection) (Fig 2C). There was a significant difference in anti-RBD IgG levels between the hybrid and vaccine only group. This pattern persisted across other time periods as well. Median (interquartile range (IQR)) of anti-RBD antibody levels among participants three months after hybrid (vaccination and infection) and the vaccine only were 227.99 (95% CI 192.67–257.44) AU/mL and 143.55 (95% CI 133.40–154.46) AU/mL respectively. Among participants 4–6 months after the hybrid and vaccine only, the levels were 155.66 (95% CI 86.73–187.29) and 59.73 (95% CI 49.55–70.63) AU/mL, respectively. For those 7–9 months after the hybrid and vaccine only, the levels were 76.11 (95% CI 45.88–95) AU/mL and 36.56 (95% CI 23.18–50.24) AU/mL, respectively. Finally, among participants beyond 10 months after the hybrid and vaccine only, the levels were 168.66 (95% CI 134.27–225.64) AU/mL and 23.11 (95% CI 5.38–33.74) AU/mL, respectively.

### Increased anti-RBD IgG levels after breakthrough infection

The study then examined whether breakthrough COVID-19 infections were associated with an improved immune response. The participants were divided into two groups: those that had experienced infections after vaccination despite being fully vaccinated (vaccine first), and those that had received vaccinations after being infected with SARS-CoV-2 (infection first).

The analysis revealed that the group with breakthrough infections after vaccination (orange dots in Fig 2D) had significantly higher levels of anti-RBD IgG antibody levels ranging from 1.7 to almost 3 times those of the group with prior infection-induced immunity across various time periods (in green in Fig 2D), showing an association between vaccine first and enhanced immune response.

### Comparison of two surveys

Two surveys were conducted with 1,064 and 1,397 participants recruited in September 2021 and March 2022, respectively. Six months after the initial survey, more people were found to be infected with COVID-19 based on both self-reported data and positive results of anti-NC antibodies. Moreover, the proportion of people with detectable anti-RBD and NC antibodies increased from 88.2% and 19.9% in September 2021 to 96.33% and 39.1% in March 2022, respectively (Table 4). Interestingly, there was an almost twofold increase in the detection rate of anti-NC antibody levels (from 8.9% to 16.5%) among participants who reported no previous history of COVID-19.

**Table 4:** The comparison of key outcome variables between the two serosurveys.

Serosurvey	I	II	P-value
Study Dates	9/13-9/17, 2021	3/1-3/3,2022	N/A
% Vaccinated (self-reported)	91.9% (95% CI 90.1–93.4%)	94.7% (95% CI 93.4–95.8%)	p = 0.007
% Infected (self-reported)	19.3% (95% CI 17–21.7%)	37.9% (95% CI 35.4–40.4%)	p<0.001
% of positive results of anti-RBD IgG	88.2% (95% CI 86.1–90%)	96.3% (95% CI 95.2–97.2%)	p<0.001
% of positive result of anti-N	19.7% (95% CI 17.5–22.2%)	39.1% (95% CI 36.6–41.7%)	p<0.001
% Unknow infection	8.9% (95% CI 7.2–11.2%)	16.5% (95% CI 14.1–19.2%)	p<0.001

### The levels of anti-RBD antibodies in the saliva correlate with the levels of antibodies in the serum

**Fig 3:**
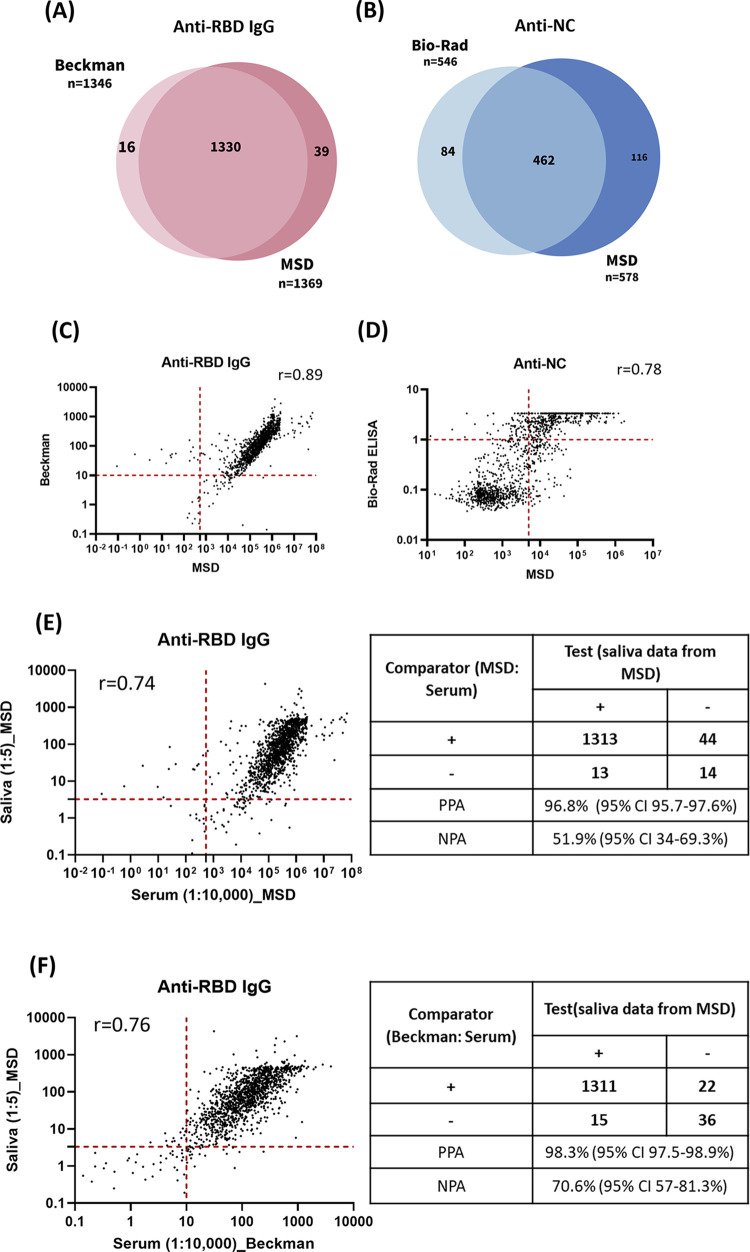
The correlation of sample types and assays. **(A)** Venn diagrams showing overlap of positive results of (A) RBD of Spike and **(B)** NC from two different assays. **(C)** Correlation between the value of anti-RBD IgG by Beckman and the MSD assay. **(D)** Correlation between the value of total anti-NC by Bio-Rad assay and the value of anti-NC IgG by the MSD assay. **(E)** Correlation of the value of anti-RBD IgG between serum and saliva samples. **(F)** Correlation between the value of anti-RBD IgG using serum samples by the Beckman assay and using saliva samples by the MSD assay. A red dotted line indicates the cutoff line. All test values equal to or greater than this line is considered positive. r is calculated using Spearman correlation.

The MSD assay was used in our study for the detection of anti-RBD antibody levels in saliva samples due to its larger dynamic detection range. In our initial investigation, we compared the MSD assay with the Beckman and Bio-Rad assays for the detection of anti-RBD and anti-NC antibodies, respectively. The Venn diagrams in Fig 3A illustrated the distribution of positive results for two different assays measuring seropositive responses to the RBD of the spike protein. In addition to the 1330 specimens that were positive for both assays, 16 and 39 specimens were exclusively positive for Beckman and MSD, respectively. The percentage of positive results for both assays for anti-RBD IgG were comparable (96.3% and 98%, respectively), which is based on the same sample population. Furthermore, Fig 3C displays the correlation (r = 0.89) between the values of anti-RBD IgG measured by the two assays.

Fig 3B showed the overlapping distribution of positive results for two different assays measuring seropositive NC. In addition to the 462 specimens that were positive for both Bio-Rad and MSD assays, 84 and 116 specimens were exclusively positive for Bio-Rad and MSD, respectively. In addition, Fig 3D displayed a positive correlation (r = 0.78) between the values of anti-NC antibody level measured by Bio-Rad and MSD assays, with somewhat more disagreement than that observed for anti-RBD IgG.

Next, we examined whether antibody levels to RBD in the saliva correlated with those measured in the serum. First, we selected 20 negative saliva samples from Serosurvey I, considering their self-reported vaccination and COVID-19 infection status, as well as the absence of any detectable anti-RBD and anti-NC antibodies, to calculate the cutoff value (average +3SD) for negative saliva samples. Next, we found a significant positive correlation (r = 0.74) between the levels of anti-RBD antibodies in paired saliva and serum samples (n = 1384). The PPA and NPA were found to be 96.8% (95% CI 95.7–97.6%) and 51.9% (95% CI 34–69.3%), respectively (Fig 3E). Furthermore, we conducted a comparison between saliva data from MSD and serum data from an EUA authorized assay. The correlation coefficient between these two data sets was 0.76. Additionally, the PPA and NPA were found to be 98.3% (95% CI 97.5–98.9%) and 70.6% (95% CI 57–81.3%), respectively (Fig 3F).

## Discussion

In a previous study, only 71.9% of people in Arizona received one dose, 60.5% received two doses, and 40.1% had a booster or additional dose as of Oct 15, 2022 [16]. By comparison, in the ASU community, 94.7% of participants self-reported at least one dose, 88.9% of participants had completed their vaccination, and 62.9% of participants had a booster of a COVID-19 vaccine. We believe that the high vaccination rate in the ASU community contributed to the low active COVID-19 positivity rate of 0.4% from saliva qPCR on the sample collection days following the Omicron wave in Arizona. The seroprevalence of anti-RBD and anti-NC antibodies after the Omicron wave in this study were found to be 96.3% (95% CI 95.2–97.2%) and 39.1% (95% CI 36.6–41.7%). We also calculated seroprevalence for both anti-RBD and anti-NC antibodies, adjusted to the reported sensitivity and specificity of the assays using previously published statistical methods [17], yielding 97.4% (95% CI 95.1–99.8%) and 38.4% (95% CI 35.7–41.1%), respectively, showing close alignment with the unadjusted values due to the high sensitivity and specificity of the assays.

Previous studies have demonstrated a decline in anti-SARS-CoV-2 antibody levels during the first six months after initial COVID-19 vaccination [18–20], though levels remained detectable for a duration of 6–8 months after the second dose of the vaccine [21–23]. However, our findings within the ASU community indicate that anti-RBD antibodies were still detectable even one year after vaccination (Fig 2B). This extended duration of detectability could be attributed to the administration of booster doses received by some participants (66.9%) after completing the standard two-dose regimen. We also observed similar results in this study, the vaccine only group showed a faster decline of anti-RBD antibodies than the hybrid group, indicating that hybrid immunity had a protective effect compared with vaccine only immunity (Fig 2C). Additionally, participants with breakthrough infections after vaccination exhibited higher levels of anti-RBD antibodies compared to those who had infections preceding vaccination (Fig 2D), consistent with our own previous serosurvey [14] and results obtained by other research groups [24–26]. We also noticed that anti-RBD antibody levels in the hybrid group increased at the last time point, possibly because some participants experienced more than one infection (Fig 2C).

The Omicron variant has been associated with milder symptoms and a higher number of asymptomatic carriers, leading to increased transmission when compared to the Delta variant [11, 13, 27]. Furthermore, studies have shown that the binding affinity of the Omicron RBD to ACE2 is slightly weaker than that of the Beta and Delta variants. [28, 29]. Moreover, a significant proportion of individuals infected with the Omicron variant were unaware of their infection [30]. These findings are consistent with the results obtained from our two serosurveys conducted during the Delta and Omicron variant waves. We observed an approximately two-fold increase in the rate of previous and unknown infections from Serosurvey I to Serosurvey II (Table 4). The results suggest that there was a substantial number of asymptomatic or mild COVID-19 cases that went undetected during the Omicron wave. An increase in the number of people with asymptomatic infection and vaccination in Serosurvey II may also explain why the median level of anti-RBD among participants 3 months after vaccination from Serosurvey II after the Omicron wave was significantly higher compared to those from Serosurvey I after the Delta wave (Fig 2A). However, we did not have information on which variants of SARS-CoV-2 participants were infected with during Serosurvey I and II, implying that some participants may have had Delta infections between Serosurvey I and II due to the overlap of the Delta and Omicron waves. Therefore, this overlap may have contributed to the higher median level of anti-RBD among participants 3 months after vaccination in Serosurvey II compared to Serosurvey I. In contrast, Blaszczuk et al. demonstrated that individuals infected with the Delta variant had higher levels of anti-RBD antibodies compared to those infected with the Omicron variant [31]. These differences could be due to differences in study design and population demographics. In the referenced study, all samples from participants underwent testing for variants of SARS-CoV-2, and blood samples were collected two months after qPCR-confirmed infection, and the population primarily consisted of individuals aged 40–50 and 70–85. Conversely, participants in our study were predominantly 18–25 years old and reported infections without knowledge of the specific variant. Additionally, anti-RBD antibody levels were measured at various times post-infection (longitudinal) compared to our cross-sectional study design.

One of the interesting findings in this study was the detection of anti-RBD antibodies in over 1000 saliva samples, with a strong positive correlation with their corresponding serum samples. This strong correlation aligns with findings from other studies that utilized different assays to compare saliva and serum samples, although with smaller sample sizes compared to ours [32–35]. We set the cutoff value for SARS-CoV-2 positive samples as more than average +3SD electrochemiluminescent signals present in 20 self-reported SARS-CoV-2 negative individuals. Their serum samples were tested negative for anti-RBD and anti-NC antibodies in EUA authorized Beckman and Bio-Rad assays, respectively. A similar approach was successfully used by Campbell et al from Meso Scale Diagnostics to test SARS-CoV-2 antibodies in saliva samples [35]. When we evaluated the MSD assay by comparing its performance with two other EUA authorized serological tests for the detection of anti-RBD and anti-NC antibody levels, the MSD assays for detecting anti-RBD and anti-NC IgG showed a strong correlation to the other assays. This observation is consistent with findings from other literature. For instance, Li et al. compared MSD assays with other clinical diagnostic assays for the detection of anti-RBD and anti-NC antibodies. They reported sensitivity and specificity values for the MSD assay for anti-RBD as 100% (95% CI 89–100%) and 94.2% (95% CI 87.1–97.5%), respectively, and for anti-NC as 100% (95% CI 89–100%) and 98.8% (95% CI 93.7–99.8%), respectively. These results indicate that MSD assays are highly compatible with other clinical assays, despite not being directly compared with the Beckman assay [36]. These findings suggest that saliva may represent a viable alternative to serum antibody testing. However, we observed a lower saliva/serum correlation for NC compared to the correlation observed for RBD (r = 0.89 vs r = 0.78), which could be due to the testing of different subtypes (total antibody vs IgG) in Bio-Rad and MSD assays (Fig 3C and 3D). Typically, antibody levels measured in saliva are approximately 10–100 times lower than those found in blood samples [37–39]. However, in our investigation, the antibody level in saliva was determined to be 100–100,000 times lower than that detected in the blood (Fig 3E and 3F). It is possible that we had higher than anticipated serum levels because of the use of the highly sensitive MDS assay in our study compared to other less sensitive serum assays employed by other laboratories. Furthermore, we identified certain saliva samples that yielded positive results while the corresponding serum samples were negative. This could potentially be due to the quality (viscosity or stickiness) of these saliva samples, potential false-positive results, also resulted in lower-than-expected NPA compared to Beckman and MSD-platform based assays.

In summary, we observed significantly higher vaccination rates within the ASU community compared to the general population of Arizona. Our seroprevalence data revealed that a high percentage of participants maintained detectable anti-RBD antibodies even a year after vaccination, especially among those who received booster doses. The study also highlighted the advantages of hybrid immunity over vaccine-only immunity in maintaining higher levels of anti-RBD antibodies. Additionally, we noted a substantial increase in the rate of previous and unknown infections during the Omicron wave, emphasizing the variant’s increased transmission and the prevalence of asymptomatic cases. The strong correlation between saliva and serum samples in detecting anti-RBD antibodies suggests that saliva could serve as an alternative medium for antibody testing.

### Limitations

Our study has several limitations. The primary limitation is that the histories of infection and vaccination relied on self-reported data, except for serological measures, posing a risk of information bias. Additionally, this population-based serosurvey was not a longitudinal cohort study, limiting its ability to provide a comprehensive picture of viral transmission dynamics and host response in the population. Moreover, although the participants were enrolled in this study, on their own without any preselection, there may be a selection bias due to their willingness to participate in the research study. Also, the data presented required grouping due to the absence of longitudinal samples. Additionally, the participants in our study were predominantly healthy individuals aged 18–25 years, which may not fully represent the seroprevalence of the entire university population.

## Supporting information

S1 Table Seroconversion by race, age, gender, employment status, and the types of vaccines from Serosurvey II (Bold indicates statistically significant differences). (DOCX)
