# Supplementary material for: The omicron variant of SARS-CoV-2 drove broadly increased seroprevalence in a public university setting
Source: PLOS Glob Public Health. 2025 Jan 3;5(1):e0003893. doi: 10.1371/journal.pgph.0003893 (PMC11698426; doi:10.1371/journal.pgph.0003893)
Supplement: S1 Table — (DOCX) [file pgph.0003893.s001.docx]

**Supplemental data for**

**The omicron variant of SARS-CoV-2 drove broadly increased seroprevalence in a public university setting**

Ching-Wen Hou^1^, Stacy Williams^1^, Guillermo Trivino-Soto^1^, Veronica Boyle^1^, David Rainford^1^, Selina Vicino^1^, Mitch Magee^1^, Yunro Chung^1,2^, Joshua LaBaer^1^, Vel Murugan^1^*

^1^ Virginia G. Piper Center for Personalized Diagnostics, Biodesign Institute, Arizona State University, Tempe, Arizona, United States of America

^2^ College of Health Solutions, Arizona State University, Phoenix, Arizona, United States of America

* vel.murugan@asu.edu

**S1 Table:** Seroconversion by race, age, gender, employment status, and the types of vaccines from Serosurvey II (Bold indicates statistically significant differences)

| **Variable** | **Comparison** | **Anti-RBD Antibody (Access SARS-CoV-2 IgG II)** | | | | **Anti-NC antibody (Platelia NC total Ab)** | | | |
| --- | --- | --- | --- | --- | --- | --- | --- | --- | --- |
|  |  | **n** | **PR** | **95% CI** | **P-value** | **n** | **PR** | **95% CI** | **P-value** |
| Race | White vs Other | 569 vs 289 | 0.99 | (0.95, 1.04) | 0.79 | 218 vs 147 | 0.87 | (0.77, 0.99) | **0.03** |
|  | Asian vs Other | 355 vs 289 | 0.98 | (0.93, 1.04) | 0.47 | 144 vs 147 | 1.05 | (0.92, 1.18) | 0.48 |
|  | White vs Asian | 569 vs 355 | 1.01 | (0.97, 1.06) | 0.58 | 218 vs 144 | 0.83 | (0.73, 0.94) | **<0.01** |
| Age | 26-40 vs 18-25 | 350 vs 594 | 1.00 | (0.96, 1.04) | 0.95 | 140 vs 276 | 0.82 | (0.71, 0.95) | **0.01** |
|  | 41+ vs 18-25 | 269 vs 594 | 1.01 | (0.95, 1.07) | 0.82 | 93 vs 276 | 1.00 | (0.83, 1.20) | 0.98 |
|  | 41+ vs 26-40 | 269 vs 350 | 1.01 | (0.96, 1.06) | 0.76 | 93 vs 140 | 1.23 | (1.01, 1.49) | **0.04** |
| Gender | Male vs Female | 509 vs 704 | 0.99 | (0.95, 1.03) | 0.53 | 216 vs 293 | 0.96 | (0.87, 1.06) | 0.46 |
| Employment Status | Student vs Employee | 728 vs 485 | 1.02 | (0.97, 1.08) | 0.39 | 325 vs 184 | 1.03 | (0.88, 1.20) | 0.70 |
|  | mRNA vaccine vs Other vaccine | 1020 vs 193 | 1.03 | (0.98, 1.08) | 0.29 | 381 vs 95 | 0.91 | (0.81, 1.01) | 0.09 |
| Vaccine Group^#^ | Unvaccinated vs Other vaccine | N/A | N/A | N/A | N/A | 33 vs 95 | 1.06 | (0.88, 1.28) | 0.55 |
|  | mRNA vaccine vs Unvaccinated | N/A | N/A | N/A | N/A | 381 vs 33 | 0.86 | (0.73, 1.01) | 0.07 |

#Unvaccinated samples were not included for the anti-RBD Antibody (Access SARS-CoV-2 IgG II)
